# Correlation between reported dengue illness history and seropositivity in rural Thailand

**DOI:** 10.1371/journal.pntd.0009459

**Published:** 2021-06-15

**Authors:** Darunee Buddhari, Kathryn B. Anderson, Gregory D. Gromowski, Richard G. Jarman, Sopon Iamsirithaworn, Butsaya Thaisomboonsuk, Taweewun Hunsawong, Anon Srikiatkhachorn, Alan L. Rothman, Anthony R. Jones, Stefan Fernandez, Stephen J. Thomas, Timothy P. Endy

**Affiliations:** 1 Department of Virology, USAMD-AFRIMS, Bangkok, Thailand; 2 State University of New York Upstate Medical University, Syracuse, New York, United States of America; 3 Walter Reed Army Institute of Research, Silver Spring, Maryland, United States; 4 Ministry of Public Health, Nonthaburi, Thailand; 5 University of Rhode Island, Providence, Rhode Island, United States of America; 6 Faculty of Medicine, King Mongkut’s Institute of Technology Ladkrabang, Bangkok, Thailand; Beijing Children’s Hospital, Capital Medical University, CHINA

## Abstract

In the latest World Health Organization (WHO) recommendation for Dengvaxia implementation, either serological testing or a person’s history of prior dengue illness may be used as supporting evidence to identify dengue virus (DENV)-immune individuals eligible for vaccination, in areas with limited capacity for laboratory confirmation. This analysis aimed to estimate the concordance between self-reported dengue illness histories and seropositivity in a prospective cohort study for dengue virus infection in Kamphaeng Phet province, a dengue-endemic area in northern Thailand. The study enrolled 2,076 subjects from 516 multigenerational families, with a median age of 30.6 years (range 0–90 years). Individual and family member dengue illness histories were obtained by questionnaire. Seropositivity was defined based on hemagglutination inhibition (HAI) assays. Overall seropositivity for DENV was 86.5% among those aged 9–45 years, which increased with age. 18.5% of participants reported a history of dengue illness prior to enrollment; 30.1% reported a previous DENV infection in the family, and 40.1% reported DENV infection in either themselves or a family member. Relative to seropositivity by HAI in the vaccine candidate group, the sensitivity and specificity of individual prior dengue illness history were 18.5% and 81.6%, respectively; sensitivity and specificity of reported dengue illness in a family member were 29.8% and 68.0%, and of either the individual or a family member were 40.1% and 60.5%. Notably, 13.4% of individuals reporting prior dengue illness were seronegative. Given the high occurrence of asymptomatic and mild DENV infection, self-reported dengue illness history is poorly sensitive for prior exposure and may misclassify individuals as ‘exposed’ when they were not. This analysis highlights that a simple, highly sensitive, and highly specific test for determining serostatus prior to Dengvaxia vaccination is urgently needed.

## Author summary

Dengue is common worldwide and more effective counter-measures are needed. The only DENV vaccine to have achieved licensure is more effective in DENV-immune individuals and is contra-indicated in DENV-naïve individuals due to safety concerns. Self-reports of prior dengue illness have been proposed to serve as a proxy for DENV exposure, due to limited resources for serological testing in many DENV-endemic locales. Here, we compare the concordance of self-reported DENV infection histories relative to serological confirmation of prior DENV exposure and report very low sensitivity and limited specificity, perhaps placing as many as one in seven individuals reporting a history of prior dengue illness at risk for an adverse outcome with receipt of Dengvaxia. We propose that reported histories of dengue illness are not sufficiently accurate to replace serological testing prior to vaccination.

## Introduction

Dengue virus (DENV) infection is the most important arboviral cause of human morbidity and mortality globally [1]_._ In the latest World Health Organization (WHO) recommendation for Dengvaxia implementation, in addition to serology testing, it is proposed that a person’s history of dengue illness could be ascertained based on medical history or medical documentation [2,3]. This pre-vaccination screening for past dengue infection, by laboratory testing or by review of medical history, is the currently recommended strategy for mitigating safety concerns associated with the administration of Dengvaxia to DENV-naïve individuals. If pre-vaccination screening is not feasible, it is recommended that vaccination without individual screening could be considered in carefully selected areas with recent documentation of seroprevalence rates of at least 80% in children aged 9 years or older. Previous epidemiological data in Kamphaeng Phet province revealed approximately 60% dengue seropositivity by 6 years of age [4,5,6] with significant clustering of DENV transmission within households [7–8]. This study aimed to identify the correlation between reported history of dengue illness by an individual or family member (as a possible proxy for prior within-home exposure) and seropositivity in a prospective cohort study for dengue virus infection.

## Methods

### Ethics statement

This study was approved by Thailand Ministry of Public Health Ethical Research Committee, Siriraj Ethics Committee on Research Involving Human Subjects, Institutional Review Board for the Protection of Human Subjects State University of New York Upstate Medical University, and Walter Reed Army Institute of Research Institutional Review Board (protocol #2119). Written informed consent was obtained from adult subjects (age ≥18 years) or the parents/guardians of child subjects (age <18 years); assent was obtained from child subjects ≥7 and <18 years of age.

### Study setting

Multigenerational family units were enrolled in a longitudinal prospective cohort study beginning in 2015 as described previously [9]. Kamphaeng Phet province (KPP), a well-established dengue-endemic area, is located in north-central Thailand about 350 km north of Bangkok, containing a population of approximately 700,000 people in a mostly rural to semi-rural setting. The medical establishment is under the service of the 410-bed Kamphaeng Phet Provincial Hospital (KPPH), health center and private clinics. Dengue diagnosis is mainly based on clinical criteria and routine blood testing, i.e., complete blood count. The Kamphaeng Phet-AFRIMS Virology Research Unit (KAVRU) has been collaborating with KPPH in dengue research for over 30 years.

### Cohort study methods

For the ongoing family cohort study, inclusion criteria included: (1) residence in Kamphaeng Phet province with no plans to move, (2) at least four family members consenting to participation including a pregnant woman (aged ≥15 years) in the third trimester, her newborn, at least one other child <18 years of age, and one grandparent. Exclusion criteria included: (1) congenital or acquired immunodeficiency, (2) immunosuppressive therapy within the preceding 6 months, (3) chronic illness that might interfere with study conduct, and (4) receipt of blood products in the previous 3 months. At the time of family unit enrollment, blood specimens and self-reported history of previous personal or familial dengue illness were collected from each individual. For very young children, dengue illness history information was elicited from their parent or guardian. Hemagglutination inhibition assays [10] were used to characterize immunity to the four DENV serotypes on enrollment. The concordance of previous reported dengue illness histories were compared against DENV serostatus as determined by hemagglutination inhibition (HAI) assays performed on specimens from enrollment into the cohort in 2015–2018.

### Data analysis

#### Defining serostatus

DENV seropositivity was defined as the presence of DENV HAI titer of >10 to any serotype at time of enrollment. DENV seronegativity was defined as DENV HAI titers of ≤10 for all four DENV serotypes.

#### Defining previous dengue illness history

A self-reported history of prior dengue illness was defined on the basis of an individual reporting having had a dengue illness diagnosed by a physician either at a hospital or clinic at any point prior to enrollment. A familial history of prior dengue illness was defined on the basis of any enrolled household member for a given participant having had a dengue illness diagnosed by a physician either at a hospital or clinic at any point prior to enrollment.

DENV seropositivity by mean age and gender were analyzed by t-test and Pearson’s Chi-Square test, respectively. Sensitivity, specificity, positive predictive value and negative predictive value, and associated receiver operating characteristic (ROC) curves, were computed by comparing DENV seropositivity (positive or negative) by HAI as the ‘gold standard’, against the three variables of self-reported history of dengue illness (present or absent), familial history of dengue illness (present or absent), and either self-reported or familial history of dengue illness (present or absent). These comparisons were restricted to those individuals aged 9–45 years old, corresponding to the age range for Dengvaxia eligibility in Thailand.

## Results

**Fig 1:**
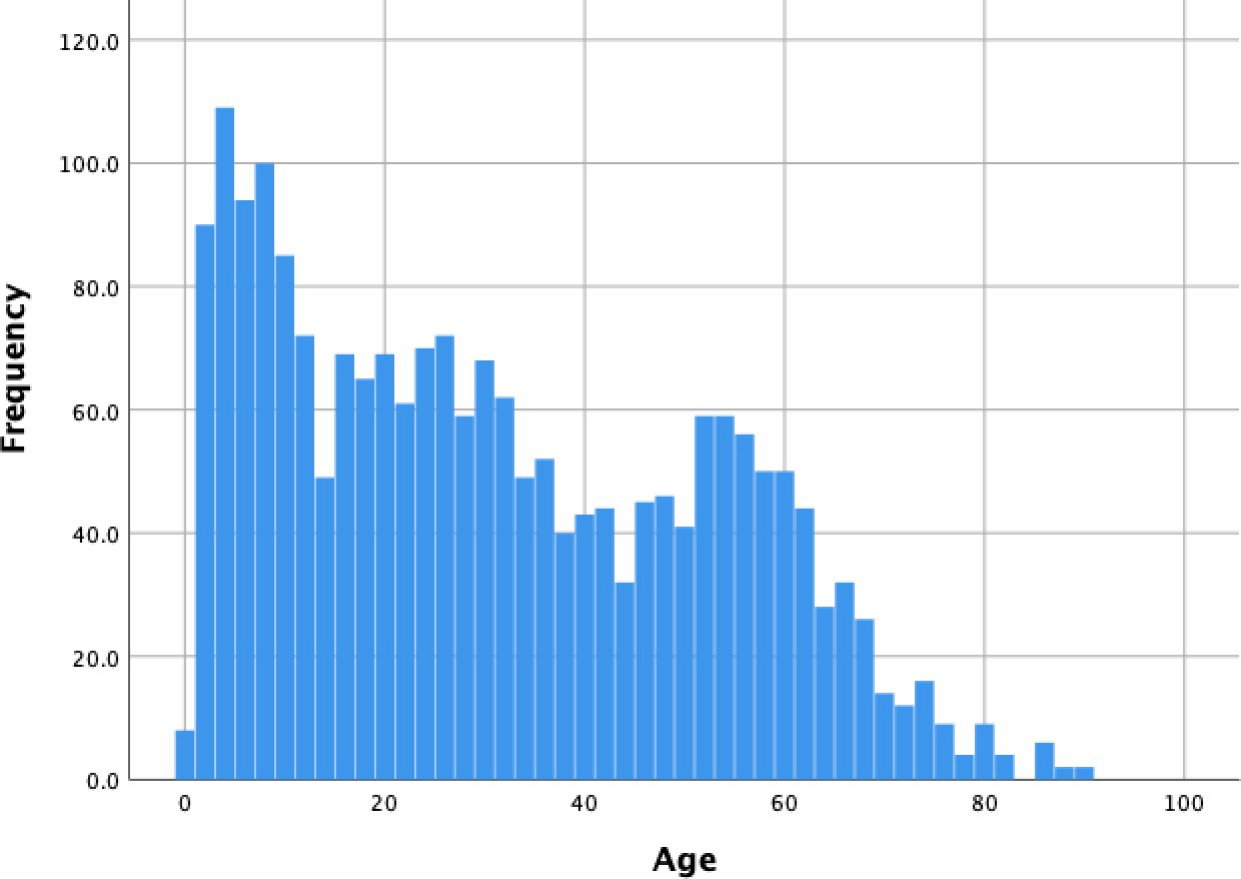
Histogram showing the age distribution of enrolled participants.

**Fig 2:**
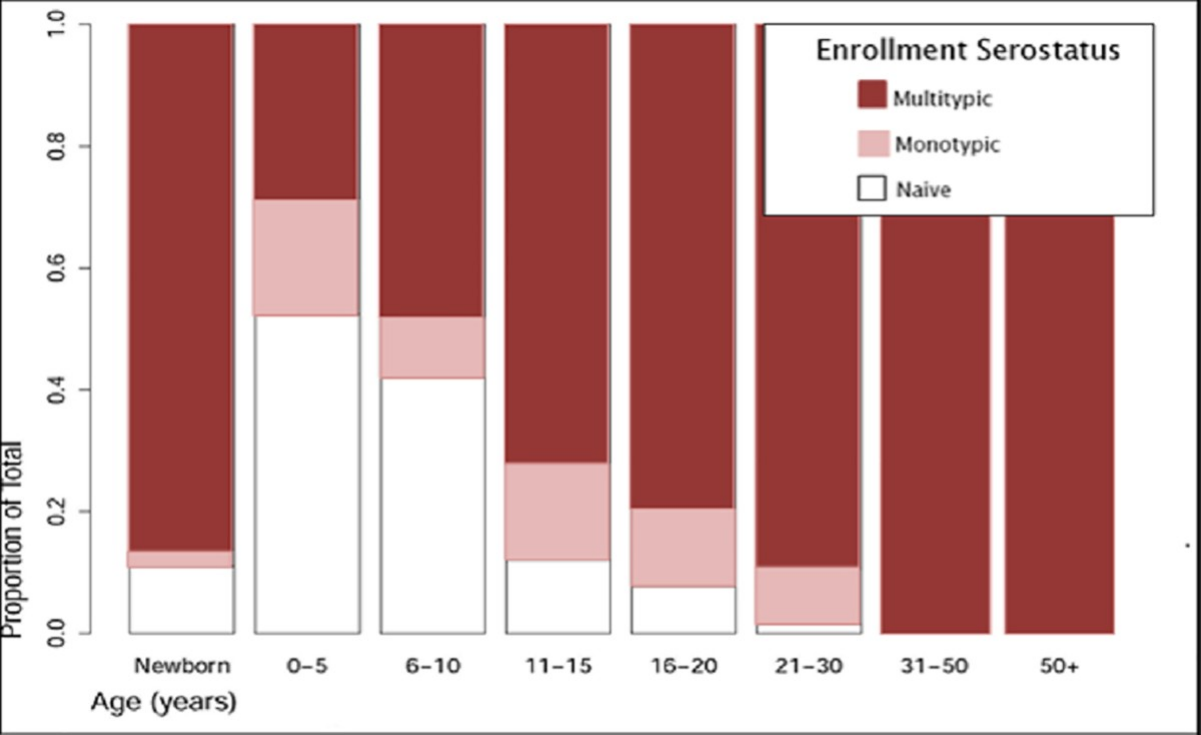
Dengue serostatus on enrollment in each age group, as determined by hemagglutination inhibition (HAI) assay. Graph show: DENV-naïve indicates HAI titers of ≤10 for all four DENV serotypes (white), ‘monotypic’ indicates an HAI titer of >10 for a single DENV serotype (pink), and ‘multitypic’ indicates HAI titers of >10 for two or more serotypes (red).

**Fig 3:**
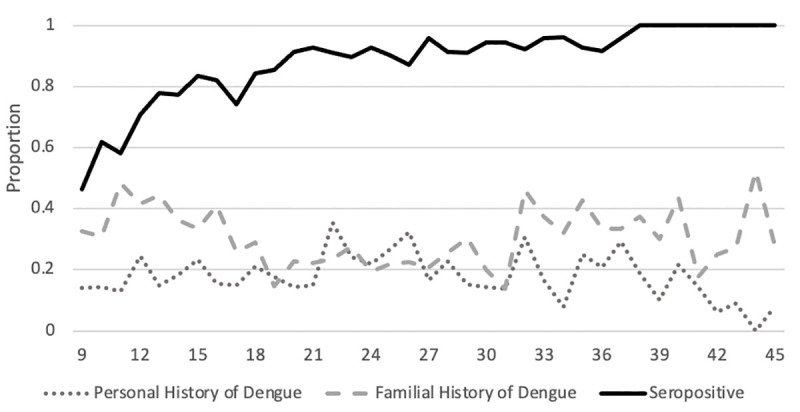
Proportions of participants aged 9–45 years with dengue seropositivity by hemagglutination inhibition assay (black), personal history of dengue (gray dots) and family history of dengue (gray dashed lines) by age.

516 families, comprising 2076 individuals, were included in this analysis with a median age of 30.6 years (range 0–90 years) (Fig 1). 63.1% of enrolled participants aged 9–45 years were female. 46.0% of children aged 9 were DENV seropositive; this proportion increased steadily with age until 100% of individuals aged 31 or older were seropositive (Fig 2). In the subset of the vaccine eligible cohort group (those aged 9–45 years), 18.5% of cohort subjects reported a personal history of prior dengue illness, 30.1% reported a familial history of prior dengue illness, and 40.1% reported either a personal history of dengue illness or illness in the family. The proportion of individuals reporting a personal history of dengue illness decreased significantly with age, while the proportion of individuals reporting a family history of dengue illness was significantly lower in young adults (aged 20–29, Fig 3).

A reported personal history of dengue illness and either a personal history of dengue illness or illness in the family were significant in terms of association with seropositivity (p = 0.002 and 0.034 by Pearson’s Chi-Square test) see Table 1. The sensitivity, specificity, PPV and NPV of self-reported history of prior dengue illness compared to seropositivity were 18.5%, 81.6%, 86.6% and 13.6% respectively. The sensitivity, specificity, PPV and NPV of a familial history of prior dengue illness were 29.8%, 68.0%, 85.6% and 13.2% respectively, and the sensitivity, specificity, PPV and NPV of either individual or familial history of prior dengue illness were 40.3%, 60.5%, 86.7% and 13.7%. There was no difference by gender in the proportion seropositive or in the proportion reporting histories of personal or familial dengue illness (p>0.05 for all).

**Table 1:** Correlation between Reported Dengue Illness History and Cohort seropositive and seronegative status.

Reported Dengue Illness History	Serostatus				p- value
	Seropositive (n = 939)		Seronegative (n = 147)		
	n	%	n	%	
Personal	174	18.5	27	18.4	**0.002**
In Family	280	29.8	47	32.0	0.280
Either Personal or Family	378	40.3	58	39.5	**0.034**

**Fig 4:**
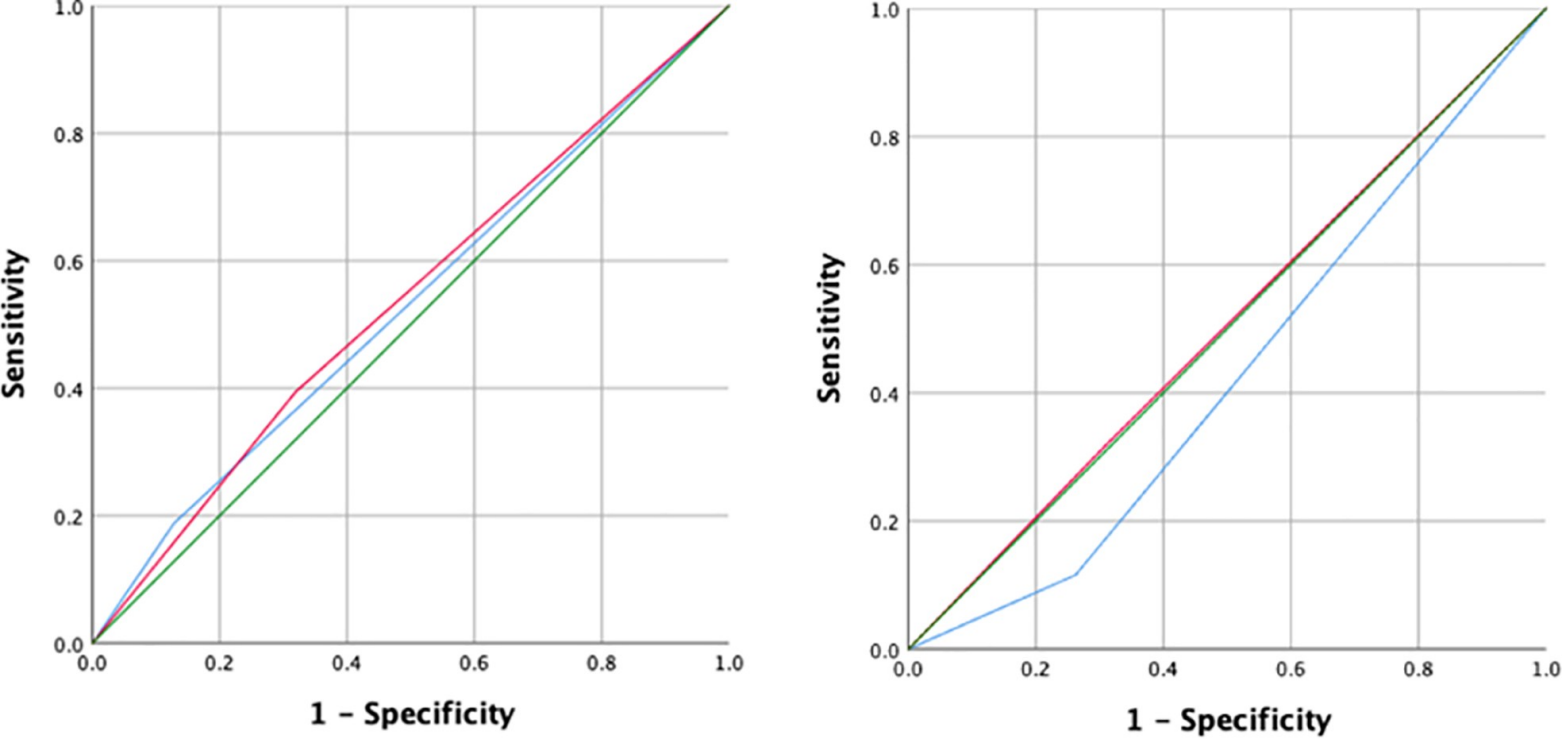
Receiver operating characteristic (ROC) curves for reported history of dengue illness as personal history (blue) and family history (red) against Hemagglutination inhibition (HAI) results. Graphs show (Left) children aged 9–17 years old (Right) adults aged 18–45 years old.

The ROC curves for reported personal and familial history against seropositivity by HAI stratified by age group, showed low sensitivity and limited specificity (Fig 4). The area under the curve (AUC) for children aged 9–17 years was 0.530 (95% confidence interval: 0.461–0.599) for personal history and 0.538 (95% CI: 0.468–0.607) for familial history of dengue illness. The AUC for adults aged 18–45 years was 0.427 (95% CI: 0.347–0.506) for personal history and 0.504 (95% CI: 0.461–0.599) for familial history of dengue illness.

## Discussion

These analyses indicate that a self-reported history of prior dengue illness may be inadequate to serve as a proxy for seropositivity. The poor sensitivity of self-report for identifying individuals with prior DENV exposure is expected, as a majority of DENV infections are subclinical [11,12]. The inclusion of familial history of dengue illness, as a proxy for possible prior exposure due to observed clustering of infections within homes, resulted in higher sensitivity but lower specificity for DENV seropositivity. Further, there may be misclassification/misdiagnosis of dengue illnesses based upon clinical criteria due to overlapping symptomatology with many other infections. Perhaps as a consequence of this diagnostic imprecision, we report that nearly one in seven reporting a prior clinical diagnosis of dengue illness was in fact seronegative. This has important implications for pre vaccination screening prior to administration of Dengvaxia, as vaccination of seronegative individuals has been associated with adverse safety outcomes [13,14,15]. Our findings underscore the critical need for a fast, simple serological assay to determine DENV serostatus prior to Dengvaxia administration. An optimal assay would be both highly sensitive, to maximize the capture of the eligible (i.e., seropositive) population and highly specific, to identify seronegative individuals who may be more likely to experience adverse effects of vaccination.

If self-reporting were used exclusively as an alternative to serological screening to identify eligible Dengvaxia recipients, this high false-negative rate would result in many missed opportunities for vaccination and reduce the cost effectiveness of vaccination programs [16,17]. More critically, the PPV of a reported dengue illness was not sufficiently high to avert vaccination of significant numbers of seronegative individuals. 13.4% of those with a self-reported history of prior dengue illness, 14.4% of those with a familial history of prior dengue illness, and 13.3% of those with either an individual or family history of prior dengue illness were seronegative, which would potentially place them at a higher risk for adverse outcomes with the Dengvaxia vaccination. These data indicate that reported histories of prior dengue illness are not sufficiently accurate to replace serological testing prior to administration of Dengvaxia.

The study is subject to limitations. Serological characterizations of DENV exposure histories are notoriously complicated by cross-reactivity between DENV serotypes and other flaviviruses [18,19,20]. The true gold standard assays for DENV serostatus focus upon quantitation of neutralizing antibodies; hemagglutination inhibition assays are used widely in the characterization of antibody responses in this and other large cohorts [21,22] as they are both fast and reasonably accurate. It is possible, however, that some individuals with prior exposures to Japanese encephalitis virus or Zika virus were misclassified as DENV-immune by HAI or that some individuals were misclassified as DENV-naïve through the use of a less-sensitive test. Additionally, recall bias may further decrease the accuracy of reported histories of dengue; this may be reflected in the decreasing proportion of individuals reporting prior dengue illness with age, as the peak age of DENV infection in the study area tends to occur during the teenage years [23,24].

Finally, these findings may not be generalizable to other locations with different dengue virus transmission and disease patterns. Thailand is a highly-endemic locale for DENV transmission and thus the reported PPV (86.6%) is higher than would be observed in areas with lower levels of exposure and seroprevalence. In such low-transmission settings, we may expect that the proportion of individuals misclassified as seropositive based upon reported history of dengue illness could be even higher. Our findings should raise concern that relying on a reported medical histories as a proxy for serological confirmation of dengue virus infection may be problematic in locations where testing capabilities are limited.
